# Supplementary material for: Epitope binning for multiple antibodies simultaneously using mammalian cell display and DNA sequencing
Source: Commun Biol. 2024 May 28;7:652. doi: 10.1038/s42003-024-06363-7 (PMC11133372; doi:10.1038/s42003-024-06363-7)
Supplement: Supplementary file 2 — Supplementary information [file 42003_2024_6363_MOESM2_ESM.pdf]

Supplementary information

Epitope binning for multiple antibodies simultaneously using mammalian cell display and DNA sequencing

Ning Lin, Kotaro Miyamoto, Takumi Ogawara, Saki Sakurai, Shinae Kizaka-Kondoh, Tetsuya Kadonosono*

School of Life Science and Technology, Tokyo Institute of Technology, Yokohama 226-8501, Japan

*Corresponding Author
Tetsuya Kadonosono
School of Life Science and Technology, Tokyo Institute of Technology, Yokohama 226-8501, Japan. Tel.: +81-45-924-5848; E-mail: tetsuyak@bio.titech.ac.jp

a

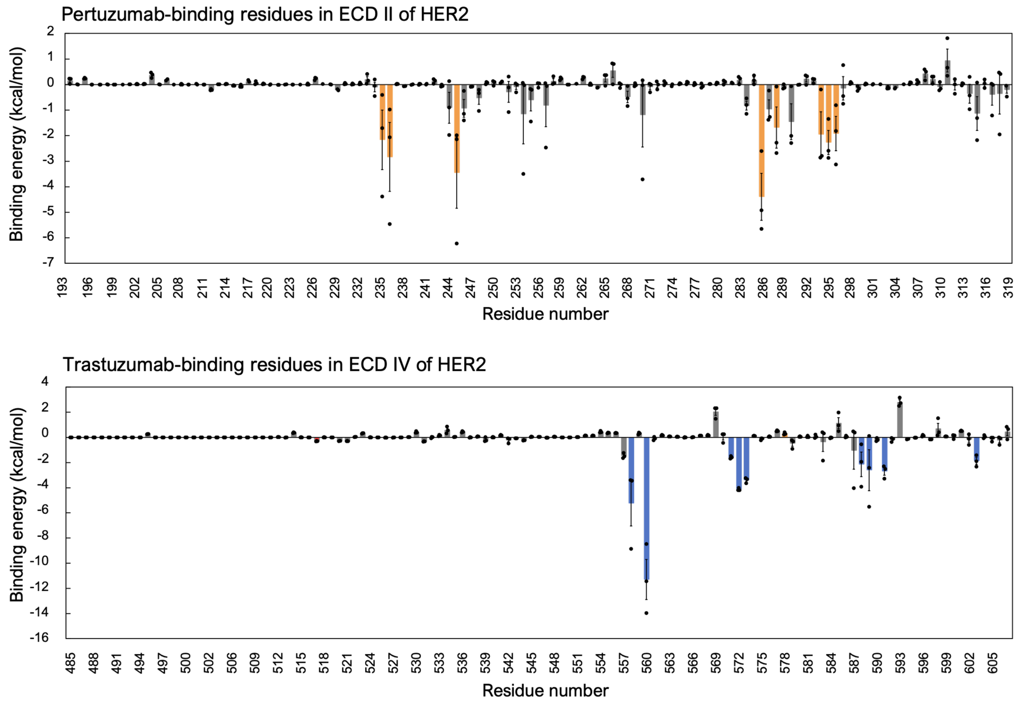

Supplementary Fig. 1. Identification of antibody-binding residues on HER2 and HER2-binding residues on antibodies

a Identification of pertuzumab- and trastuzumab-binding residues on HER2. The binding free energy of each residue on HER2 to pertuzumab and trastuzumab was calculated by MD and is shown as the mean ± SEM. Bars in orange and blue represent residues with binding free energy values of < -1.5 kcal/mol as epitope-constituting residues for pertuzumab and trastuzumab, respectively. n = 3.

b

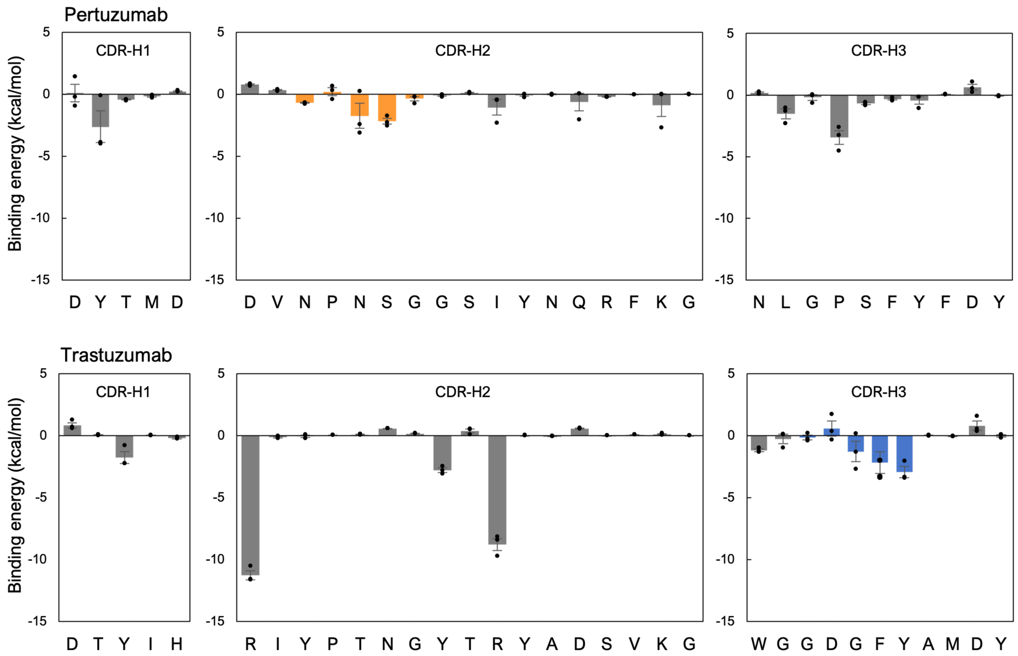

Supplementary Fig. 1 (continued). Identification of antibody-binding residues on HER2 and HER2-binding residues on antibodies

b Identification of HER2-binding residues on pertuzumab and trastuzumab. The binding free energy of each residue on complementarity-determining regions (CDRs) of pertuzumab and trastuzumab to HER2 was calculated by MD and is shown as the mean ± SEM. Bars in orange and blue represent residues for alanine substitution to generate mutant scFv(Per) and mutant scFv(Tra), respectively. n = 3.

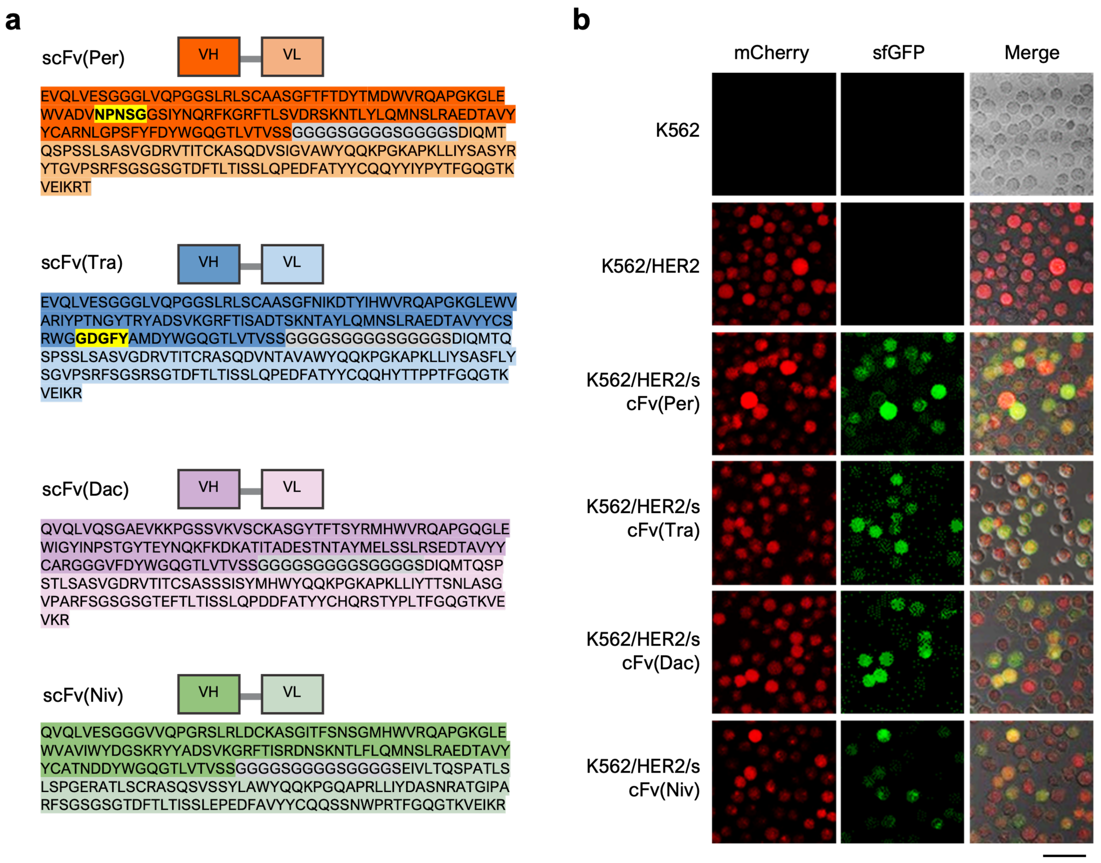

Supplementary Fig. 2. Establishment of qAb-displaying cells

a Structural diagram and the sequences of pertuzumab scFv [scFv(Per)], trastuzumab scFv [scFv(Tra)], daclizumab scFv [scFv(Dac)], and nivolumab scFv [scFv(Niv)]. The sequence of the variable domains of the heavy (VH) and light (VL) chains are highlighted by thick and thin colors, respectively. The linker sequence is also shown. The sequences highlighted in yellow in scFv(Per) and scFv(Tra) are residues for alanine substitution to generate mutant scFv(Per) and mutant scFv(Tra), respectively. **b** mCherry and sfGFP expression in K562/HER2/scFv(Per), K562/HER2/scFv(Tra), K562/HER2/scFv(Dac), and K562/HER2/scFv(Niv) cells. Scale bar = 50 μm.

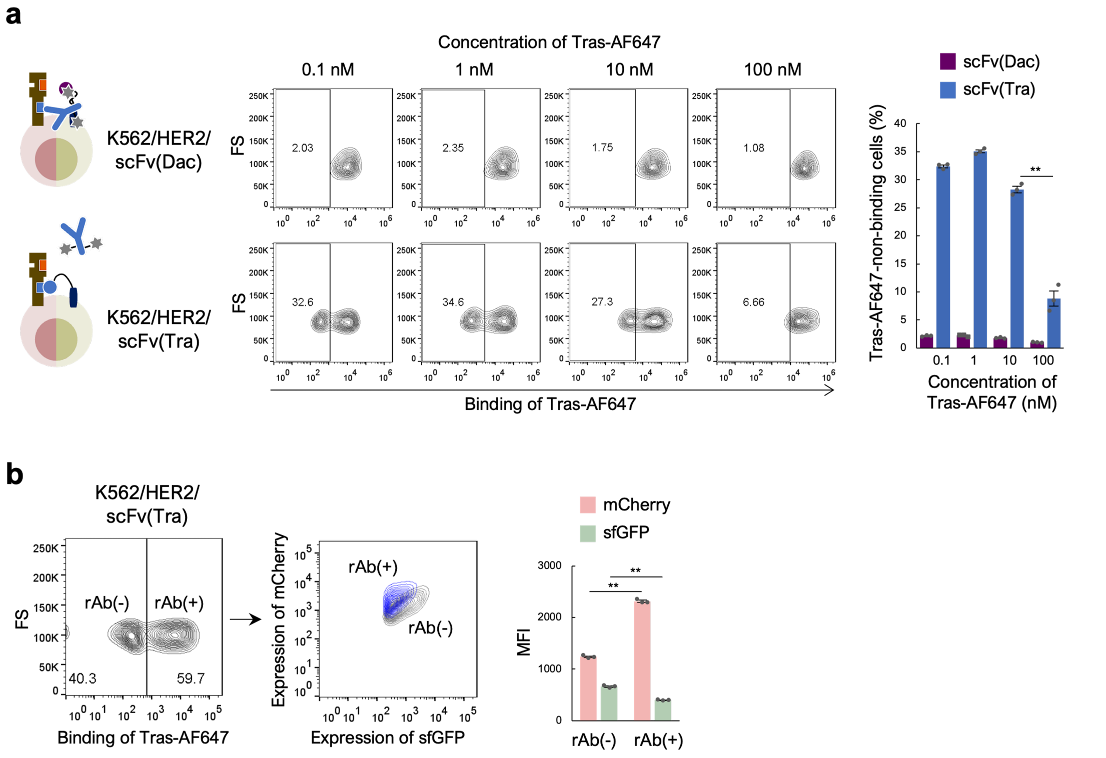

Supplementary Fig. 3. Effects of the Tras-AF647 concentration and the balance between qAb and HER2 expression levels on evaluation sensitivity

a Evaluation of epitope similarity between qAb and various concentrations of Tras-AF647. K562/HER2/scFv(Dac) and K562/HER2/scFv(Tra) cells were incubated with 0.1–100 nM Tras-AF647 prior to FCM analysis. Representative contour plots are shown. Percentage of Tras-AF647-non-binding cells calculated using FCM plots from three independent experiments is also shown as the mean ± SEM. $^{**}p < 0.0001$ (Tukey's multiple comparisons test). n = 3. **b** Expression levels of scFv(Tra) and HER2 in Tras-AF647-non-binding [rAb(-)] and Tras-AF647-binding [rAb(+)] cell populations. K562/HER2/scFv(Tra) cells were incubated with 1 nM Tras-AF647 prior to FCM analysis and the expression levels of scFv(Tra) and HER2 were estimated according to the fluorescent intensity of sfGFP and mCherry, respectively. Representative contour plots are shown. MFI values of sfGFP and mCherry in rAb(-) and rAb(+) cell populations calculated using FCM plots from three independent experiments are shown as the mean ± SEM. $^{**}p < 0.0001$ (*t*-test). n = 3.

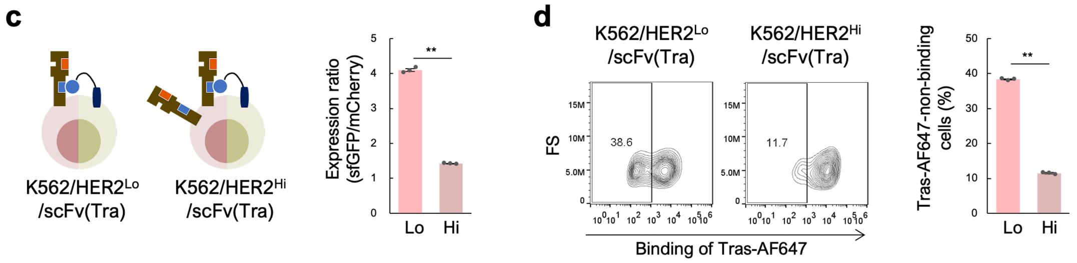

Supplementary Fig. 3 (continued). Effects of the Tras-AF647 concentration and the balance between qAb and HER2 expression levels on evaluation sensitivity

c Balance between scFv(Tra) and HER2 expression levels in K562/HER2^{Lo}/scFv(Tra) (Lo) and K562/HER2^{Hi}/scFv(Tra) (Hi) cells. Expression levels of scFv(Tra) and HER2 were estimated according to the fluorescent intensity of sfGFP and mCherry on FCM analysis, respectively, and the relative expression ratio was calculated using the MFI values of sfGFP and mCherry from three independent experiments. The mean ± SEM is shown. **$p < 0.0001$ (t-test). n = 3. **d** Epitope similarity evaluation between qAb and Tras-AF647 using cells with different HER2 expression levels. K562/HER2^{Lo}/scFv(Tra) (Lo) and K562/HER2^{Hi}/scFv(Tra) (Hi) cells were incubated with 1 nM Tras-AF647 prior to FCM analysis. Representative contour plots are shown. Percentage of Tras-AF647-non-binding cells calculated using FCM plots from three independent experiments is also shown as the mean ± SEM. **$p < 0.0001$ (t-test). n = 3.

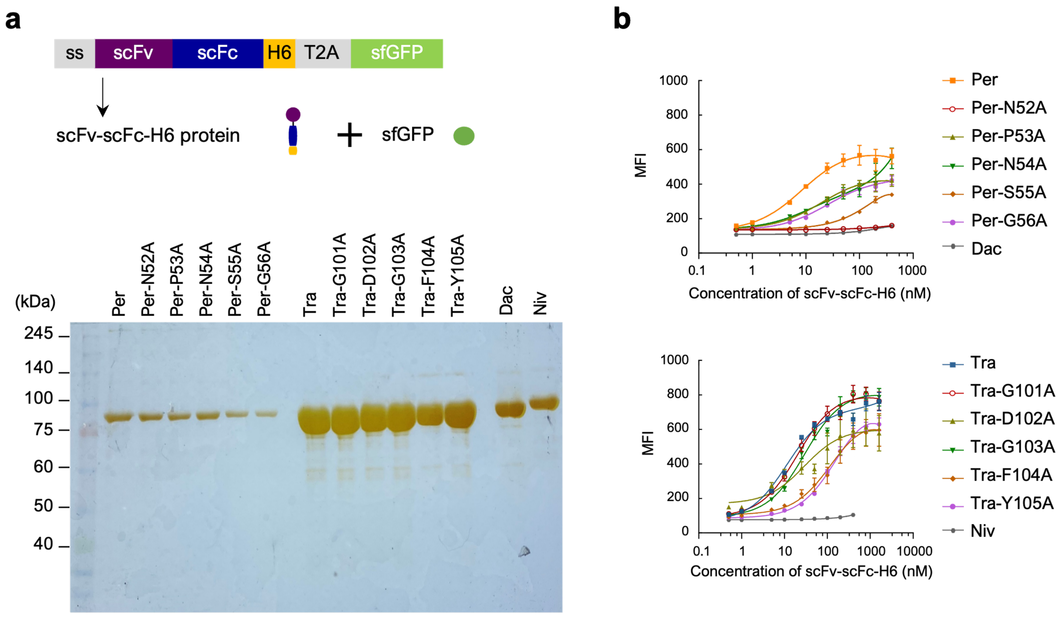

Supplementary Fig. 4. HER2-binding affinity of scFvs

a Preparation of scFc- and His6 tag peptide (H6)-fused scFv (scFv-scFc-H6) proteins. Diagram of cDNA construct in the plasmids pCSII/scFv-scFc for the expression of scFv-scFc-H6 proteins that are secreted extracellularly. Purity of purified scFv-scFc-H6 proteins. Fourteen scFvs, namely Per, Per-N52A, Per-P53A, Per-N54A, Per-S55A, Per-G56A, Tra, Tra-G101A, Tra-D102A, Tra-G103A, Tra-F104A, Tra-Y105A, Dac, and Niv, were expressed as scFv-scFc-H6 proteins and purified using affinity beads. The purified protein solutions were separated by polyacrylamide gel electrophoresis and visualized with silver staining. The size of all fusion proteins was approximately 80 kDa. **b** Binding of scFv-scFc-H6 proteins to HER2. K562/HER2 cells were incubated with various concentrations of scFv-scFc-H6 proteins prior to FCM analysis using AF488-conjugated anti-His tag antibody. Representative plots of MFI values against the concentration of scFv-scFc-H6 proteins are shown. Plots were fitted using a one-site total nonlinear regression curve fitting model in GraphPad Prism 10 and this was used for K_D calculation. n = 3.

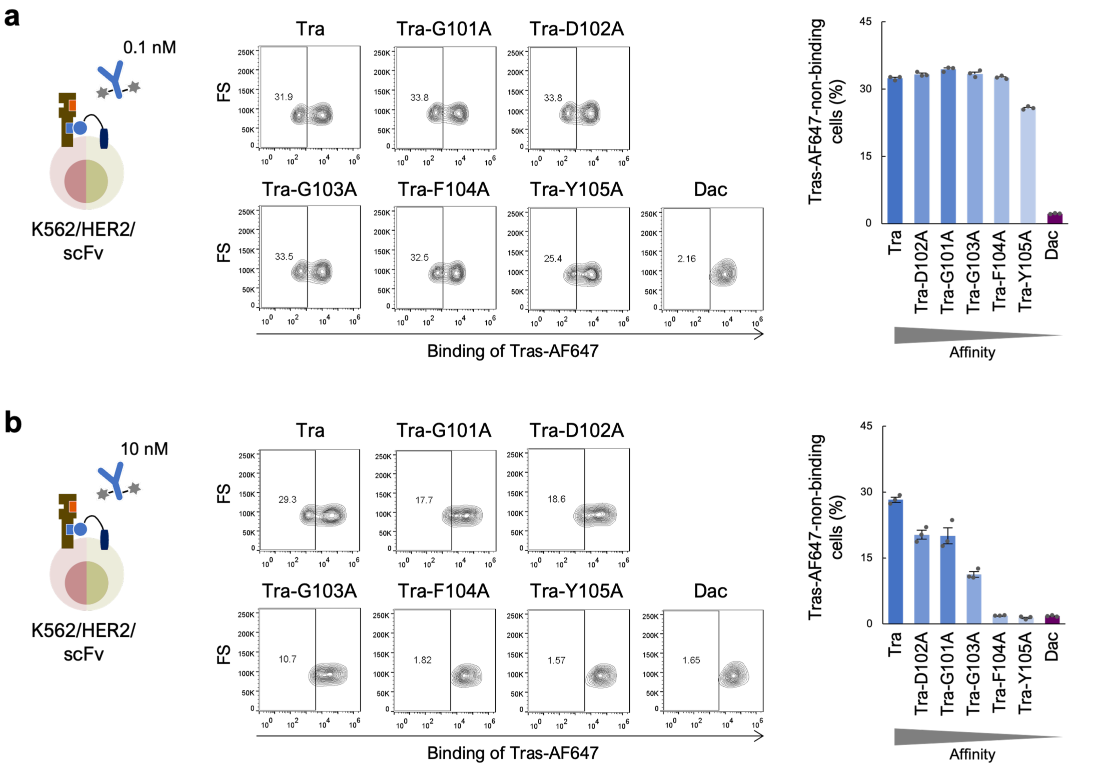

Supplementary Fig. 5. Evaluation of Tra mutants with various HER2-binding affinities

a Epitope similarity evaluation between qAb and Tras-AF647 at 0.1 nM. K562/HER2 cells displaying Tra, Tra mutants, and Dac were incubated with 0.1 nM Tras-AF647 prior to FCM analysis. Representative contour plots are shown. The percentage of Tras-AF647-non-binding cells calculated using FCM plots from three independent experiments is also shown as the mean ± SEM. n = 3. **b** Epitope similarity evaluation between qAb and Tras-AF647 at 10 nM. K562/HER2 cells displaying Tra, Tra mutants, and Dac were incubated with 10 nM Tras-AF647 prior to FCM analysis. Representative contour plots are shown. The percentage of Tras-AF647-non-binding cells calculated using FCM plots from three independent experiments is also shown as the mean ± SEM. n = 3.

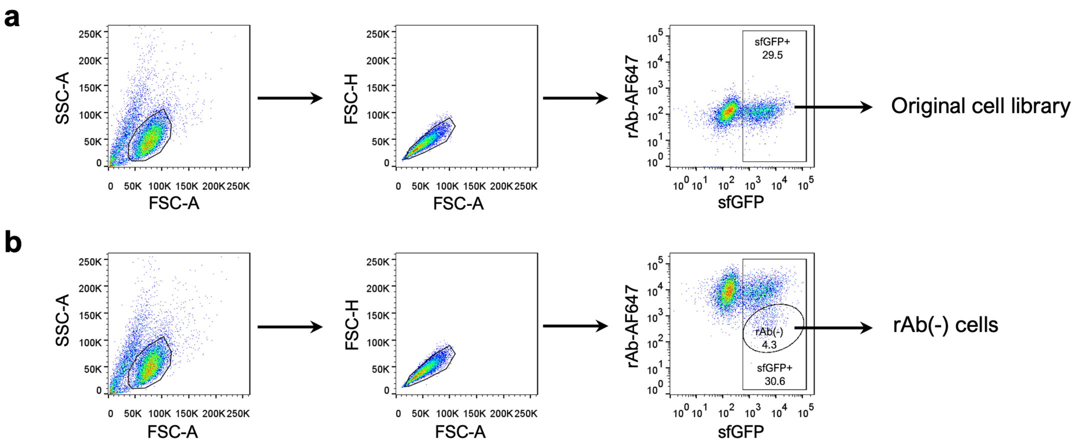

Supplementary Fig. 6. Gating strategy for sorting library cells and rAb(-) cells

a The sfGFP-positive (sfGFP+) cells were gated and sorted as the original library from the mixed cells. **b** Mixed cells were reacted with rAb, followed by gating sfGFP+ cells for sorting rAb(-) cells.

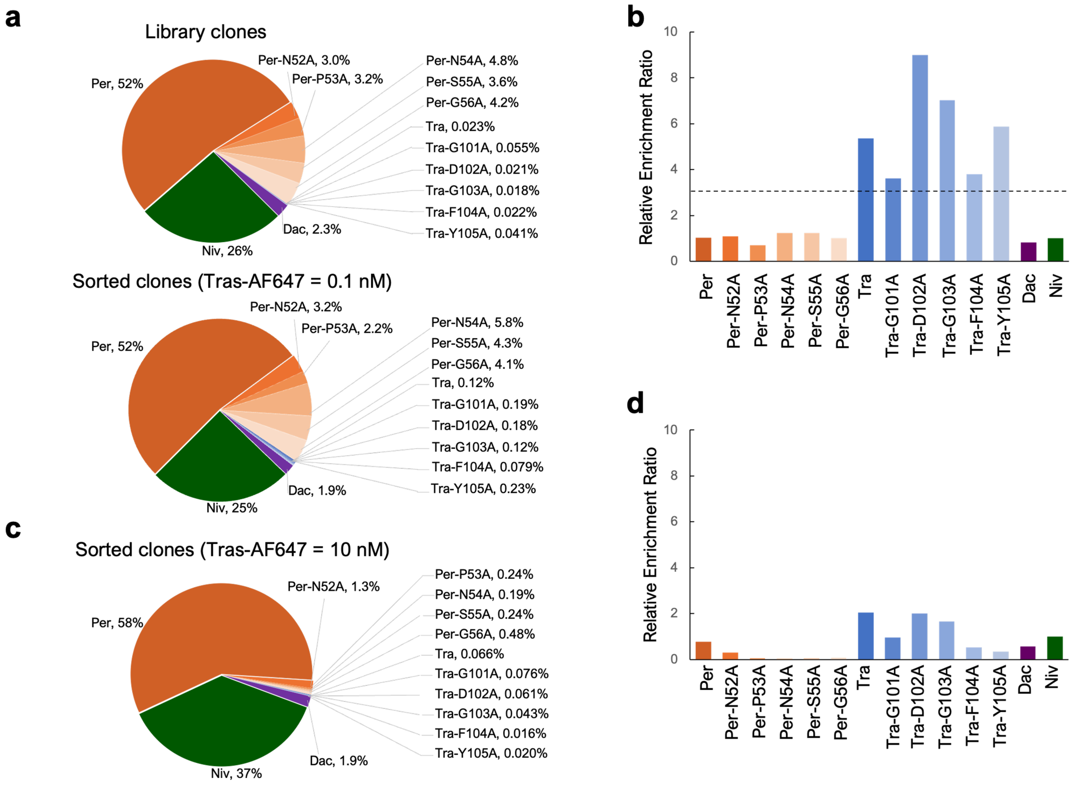

Supplementary Fig. 7. Epitope Binning-seq for various qAbs using Tras-AF647

a Occupancy of each scFv before and after sorting using Tras-AF647 at 0.1 nM. Cell library was incubated with 0.1 nM Tras-AF647 and the rAb(-) cell population was collected for NGS analysis. **b** Relative enrichment ratio of each scFv calculated from the occupancy in **a**. **c** Occupancy of each scFv after sorting using Tras-AF647 at 10 nM. The same cell library in **a** was incubated with 10 nM Tras-AF647 and the rAb(-) cell population was collected for NGS analysis. **d** Relative enrichment ratio of each scFv calculated from the occupancy in **a** (library cells) and **c**.
