# Supplementary material for: Epitope binning for multiple antibodies simultaneously using mammalian cell display and DNA sequencing
Source: Commun Biol. 2024 May 28;7:652. doi: 10.1038/s42003-024-06363-7 (PMC11133372; doi:10.1038/s42003-024-06363-7)
Supplement: Supplementary file 5 — Reporting summary [file 42003_2024_6363_MOESM5_ESM.pdf]

nature portfolio

Corresponding author(s): Tetsuya Kadonosono
Last updated by author(s): May 9, 2024

Reporting Summary

Nature Portfolio wishes to improve the reproducibility of the work that we publish. This form provides structure for consistency and transparency in reporting. For further information on Nature Portfolio policies, see our Editorial Policies and the Editorial Policy Checklist.

Statistics

For all statistical analyses, confirm that the following items are present in the figure legend, table legend, main text, or Methods section.

n/a	Confirmed	
☐	☒	The exact sample size (*n*) for each experimental group/condition, given as a discrete number and unit of measurement
☒	☐	A statement on whether measurements were taken from distinct samples or whether the same sample was measured repeatedly
☐	☒	The statistical test(s) used AND whether they are one- or two-sided<br>*Only common tests should be described solely by name; describe more complex techniques in the Methods section.*
☒	☐	A description of all covariates tested
☒	☐	A description of any assumptions or corrections, such as tests of normality and adjustment for multiple comparisons
☐	☒	A full description of the statistical parameters including central tendency (e.g. means) or other basic estimates (e.g. regression coefficient) AND variation (e.g. standard deviation) or associated estimates of uncertainty (e.g. confidence intervals)
☒	☐	For null hypothesis testing, the test statistic (e.g. *F*, *t*, *r*) with confidence intervals, effect sizes, degrees of freedom and *P* value noted<br>*Give P values as exact values whenever suitable.*
☒	☐	For Bayesian analysis, information on the choice of priors and Markov chain Monte Carlo settings
☒	☐	For hierarchical and complex designs, identification of the appropriate level for tests and full reporting of outcomes
☒	☐	Estimates of effect sizes (e.g. Cohen's *d*, Pearson's *r*), indicating how they were calculated

Our web collection on statistics for biologists contains articles on many of the points above.

Software and code

Policy information about availability of computer code

Data collection	iCyt ec800, FACSCanto II for flow cytometry data acquisition, FACSAria III for flow cytometry data acquisition and cell sorting. Zeiss LSM780 and ZEN 3.4 for taking micrograph.
Data analysis	FlowJo 10.8.1 for flow cytometry analysis. GraphPad Prism 10 for statistical analysis.

For manuscripts utilizing custom algorithms or software that are central to the research but not yet described in published literature, software must be made available to editors and reviewers. We strongly encourage code deposition in a community repository (e.g. GitHub). See the Nature Portfolio guidelines for submitting code & software for further information.

Data

Policy information about availability of data

All manuscripts must include a data availability statement. This statement should provide the following information, where applicable:
- Accession codes, unique identifiers, or web links for publicly available datasets
- A description of any restrictions on data availability
- For clinical datasets or third party data, please ensure that the statement adheres to our policy

All data generated during this study are available from the corresponding author on reasonable request.

Human research participants

Policy information about studies involving human research participants and Sex and Gender in Research.

Reporting on sex and gender	*Use the terms sex (biological attribute) and gender (shaped by social and cultural circumstances) carefully in order to avoid confusing both terms. Indicate if findings apply to only one sex or gender; describe whether sex and gender were considered in study design whether sex and/or gender was determined based on self-reporting or assigned and methods used. Provide in the source data disaggregated sex and gender data where this information has been collected, and consent has been obtained for sharing of individual-level data; provide overall numbers in this Reporting Summary. Please state if this information has not been collected. Report sex- and gender-based analyses where performed, justify reasons for lack of sex- and gender-based analysis.*
Population characteristics	*Describe the covariate-relevant population characteristics of the human research participants (e.g. age, genotypic information, past and current diagnosis and treatment categories). If you filled out the behavioural & social sciences study design questions and have nothing to add here, write "See above."*
Recruitment	*Describe how participants were recruited. Outline any potential self-selection bias or other biases that may be present and how these are likely to impact results.*
Ethics oversight	*Identify the organization(s) that approved the study protocol.*

Note that full information on the approval of the study protocol must also be provided in the manuscript.

Field-specific reporting

Please select the one below that is the best fit for your research. If you are not sure, read the appropriate sections before making your selection.

☒ Life sciences ☐ Behavioural & social sciences ☐ Ecological, evolutionary & environmental sciences

For a reference copy of the document with all sections, see nature.com/documents/nr-reporting-summary-flat.pdf

Life sciences study design

All studies must disclose on these points even when the disclosure is negative.

Sample size	All experimental data were collected from three independent experiments and subjected to Tukey's multiple comparisons test or Student's t test.
Data exclusions	No data was excluded from the analyses.
Replication	All experiments, with the exception of for next-generation sequencing, have been confirmed to be reproducible by three independent experiments, and all attempts at replications were successful.
Randomization	Randomization does not apply to this research, as our focus is on observing and comparing outcomes between different conditions without the involvement of grouping.
Blinding	Blinding for the experiments in this study is not necessary as the focus of the study is on system establishment and characterization, without involving in the investigation of biological effects.

Reporting for specific materials, systems and methods

We require information from authors about some types of materials, experimental systems and methods used in many studies. Here, indicate whether each material, system or method listed is relevant to your study. If you are not sure if a list item applies to your research, read the appropriate section before selecting a response.

Materials & experimental systems

n/a	Involved in the study
☐	☒ Antibodies
☐	☒ Eukaryotic cell lines
☒	☐ Palaeontology and archaeology
☒	☐ Animals and other organisms
☒	☐ Clinical data
☒	☐ Dual use research of concern

Methods

n/a	Involved in the study
☒	☐ ChIP-seq
☐	☒ Flow cytometry
☒	☐ MRI-based neuroimaging

Antibodies

Antibodies used	pertuzumab (supplier: MedChemExpress, catalog number: HY-P9912), trastuzumab (Herceptin Intravenous Infusion, supplier: Chugai pharmaceutical, approval number: 21300AMY00128), Alexa Fluor 488 anti-human HER2 antibody (clone: 24D2, supplier: BioLegend, catalog number: 324410), Alexa Fluor 647 anti-His tag antibody (clone: OGHis, supplier: MBL Life Science, catalog number: D291-A64), Alexa Fluor 488 anti-His Tag antibody (clone: J099B12, supplier: BioLegend, catalog number: 652509)
Validation	Pertuzumab and trastuzumab have been well validated for targeting the human HER2, and their application in flow cytometry has been proven effective in published research. Other fluorescently-conjugated antibodies used were purchased with confirmation on the manufacture's website that they were validated for tareting either the human HER2 or His tag and suitability for flow cytometry application.

Eukaryotic cell lines

Policy information about cell lines and Sex and Gender in Research

Cell line source(s)	The human chronic myelogenous leukemia cell line K562 and subclones of the human embryonic kidney cell lines HEK293, HEK293T, and the Lenti-X 293T cell line were obtained from JCRB Cell Bank , RIKEN Bio-Resource Center, and Clontech , respectively. K562/HER2 and K562/HER2/scFv cells were established through lentiviral transduction.
Authentication	We did not conduct any verification procedures ourselves.
Mycoplasma contamination	All cell lines were regularly tested negative for mycoplasma contamination.
Commonly misidentified lines (See ICLAC register)	No commonly misidentified cell lines were used.

Flow Cytometry

Plots

Confirm that:

☒ The axis labels state the marker and fluorochrome used (e.g. CD4-FITC).

☒ The axis scales are clearly visible. Include numbers along axes only for bottom left plot of group (a 'group' is an analysis of identical markers).

☒ All plots are contour plots with outliers or pseudocolor plots.

☒ A numerical value for number of cells or percentage (with statistics) is provided.

Methodology

Sample preparation	To validate surface expression, evaluate epitope similarity, and conduct cell sorting, the specified cells or cell library were stained with indicated antibodies for 1 h at 4°C, followed by flow cytometric analysis or cell sorting. For cells used for expression validation, excess antibodies were removed by washing before analysis. For binding affinity measurement, K562/HER2 cells were incubated with various concentrations of purified scFv-scFc for 90 min at 4°C. After removal of excess scFv by washing, the cells were stained with AF488-conjugated anti-His Tag antibody, followed by washing and flow cytometry analysis. FACS buffer (3% FBS in PBS) was used for antibody dilution, cell washing, and cell resuspension.
Instrument	iCyt ec800, FACSCanto II, FACSAria III
Software	FlowJo ver.10.8.1
Cell population abundance	In the cell sorting experiment, the percentage of rAb-negative cells in singlets ranged from 3% to 4%. We specifically gated singlet cells that were sfGFP-positive and sorted those that were both sfGFP-positive and rAb-negative.

Gating strategy

From all data points, debris was excluded with FCS-A and SSC-A parameters, singlets were gated based on FSC-A and FSC-H, and target cells were gated with fluorescently-labeled antibodies.

☒ Tick this box to confirm that a figure exemplifying the gating strategy is provided in the Supplementary Information.
